# Chitosan Oligosaccharides Attenuates Oxidative-Stress Related Retinal Degeneration in Rats

**DOI:** 10.1371/journal.pone.0077323

**Published:** 2013-10-14

**Authors:** I-Mo Fang, Chang-Hao Yang, Chung-May Yang, Muh-Shy Chen

**Affiliations:** 1 Department of Ophthalmology, Taipei City Hospital Zhongxiao Branch, Taipei, Taiwan; 2 Department of Ophthalmology, National Taiwan University Hospital, Taipei, Taiwan; University of Rochester, United States of America

## Abstract

This study investigated the therapeutic potential and mechanisms of chitosan oligosaccharides (COS) for oxidative stress-induced retinal diseases. Retinal oxidative damage was induced in Sprague-Dawley rats by intravitreal injection of paraquat (PQ). Low-dose (5 mg/kg) or high-dose (10 mg/kg) COS or PBS was intragastrically given for 14 days after PQ injection. Electroretinograms were performed to determine the functionality of the retinas. The surviving neurons in the retinal ganglion cell layer and retinal apoptosis were determined by counting Neu N-positive cells in whole-mounted retinas and TUNEL staining, respectively. The generation of reactive oxygen species (ROS) was determined by lucigenin- and luminol-enhanced chemiluminescence. Retinal oxidative damages were assessed by staining with nitrotyrosine, acrolein, and 8-hydroxy-2'-deoxyguanosine (8-OHdG). Immunohistochemical studies were used to demonstrate the expression of nuclear factor-kappa B (NF-κB) p65 in retinas. An in vitro study using RGC-5 cells was performed to verify the results. We demonstrated COS significantly enhanced the recovery of retinal function, preserved inner retinal thickness, and decreased retinal neurons loss in a dose-dependent manner. COS administration demonstrated anti-oxidative effects by reducing luminol- and lucigenin-dependent chemiluminenscense levels and activating superoxide dismutase and catalase, leading to decreased retinal apoptosis. COS markedly reduced retinal NF-κB p65. An in vitro study demonstrated COS increased IκB expression, attenuated the increase of p65 and thus decreased NF-κB/DNA binding activity in PQ-stimulated RGC-5 cells. In conclusion, COS attenuates oxidative stress-induced retinal damages, probably by decreasing free radicals, maintaining the activities of anti-oxidative enzymes, and inhibiting the activation of NF-κB.

## Introduction

The eye is a unique organ because of its constant exposure to oxidative stress such as radiation, atmospheric oxygen, environmental chemicals, and physical abrasion. Many ocular diseases, including glaucoma, age-related macular degeneration, and diabetic retinopathy are caused by oxidative stress [1-4]. These diseases are the leading causes of blindness worldwide. Current available treatments are conservative, aiming to prevent sequent complications such as neovascularization formation or vitreous hemorrhage. It is therefore important to investigate new therapeutic methods to treat these diseases. 

Chitosan oligosaccharides (COS), the hydrolyzed product of chitosan, is a mixture of oligomers of β-1,4-linked d-glucosamine residues and is abundant in the exoskeleton of crustaceans and in cell walls of fungi and insects [5]. Because it is readily soluble in water due to its shorter chain and easily absorbed through the intestine, COS can quickly enter the bloodstream and exert systemic therapeutic effects. COS has been used as one of the constituent in many healthy foods or dietary supplements due to its various biological activities, including hypocholesterolemic activity, antitumor, antimicrobial, immune-enhancing, and anti-apoptotic effects [6-9]. Recently, increasing attention has now been paid to the use of the COS as antioxidants. COS has also been shown to induce intracellular GSH level, and exert protective effects on oxidative damages in various cell lines [10-12]. Although the beneficial effects of COS on oxidative damages in vitro have been studied, the effects of COS on an animal model of experimentally induced retinal oxidative damages have not yet been explored.

Nuclear transcription factor κB (NF-κB) is one of the major signal transduction pathways that activates in response to oxidative stress and regulates the expression of a variety of genes involved in inflammatory responses, cell proliferation, oxidative stress, and apoptosis [13]. In resting cells, NF-κB is maintained in the cytosol as a heterodimer in complex with its inhibitory protein, IκB. When cells are stimulated, IκB is phosphorylated by IκB kinase and degraded. This phosphorylation disassociates NF-κB from IκB and allows NF-κB to translocate to the nucleus, causing activation of NF-κB-dependent genes [14]. NF-κB is an important regulator of oxidative stress. The transcription of NF-κB-dependent genes influences the levels of reactive oxygen species (ROS) in the cell, and in turn, the levels of NF-κB activity are also regulated by the levels of ROS [15]. 

Paraquat (PQ) is a bipyridyl herbicide capable of generating oxygen radicals through the redox cycling mechanism. Cingolani et al. demonstrated that intravitreous injection of paraquat induced dose-dependent oxidative damage in the diffuse retinas of C57BL/6 mice. The oxidative damage resulted in cell death by apoptosis, causing morphologic changes in the retina and reduced retinal function as assessed by electroretinogram (ERG) [16]. Since the eye is a relatively confined compartment, intravitreous paraquat injection is relatively safe for local exposure of the retina with negligible systemic side effects. Therefore, intravitreous paraquat injection provides a good model of oxidative damage-induced retinal degeneration occurring over a short time period. 

In this study, we evaluate the therapeutic effects of COS in a rat model of oxidative stress-induced retinal disease. Furthermore, possible mechanisms by which COS exerted its action were studied in vivo and in RGC-5 cells.

## Results

### Effects of COS on electroretinogram

**Figure 1:**
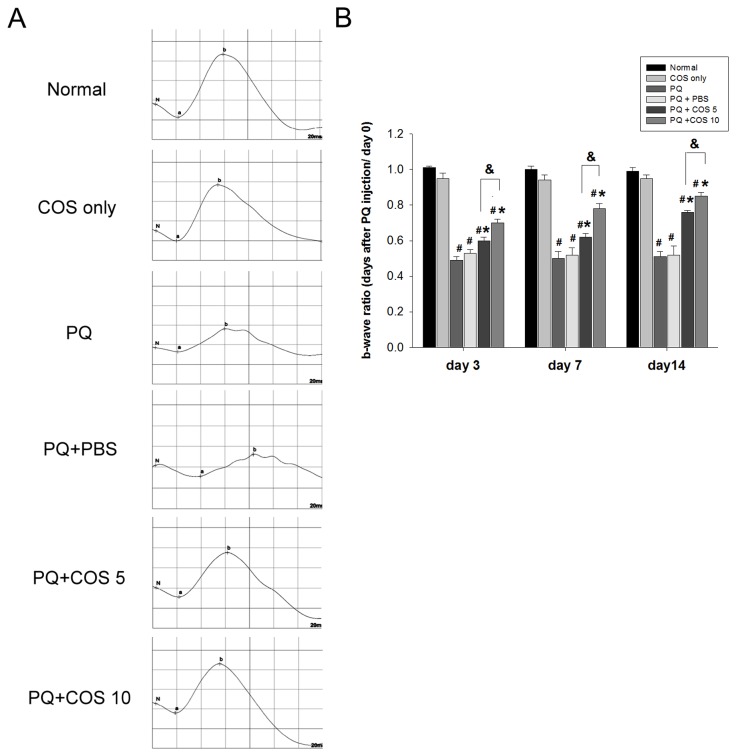
Chitosan oligosaccharides (COS) treatment attenuated reduction of electroretinogram (ERG) b- wave amplitudes. (A). Individual typical ERG records of the six groups 14 days after PQ injection. (B). Mean ERG b-wave amplitude was measured at day 3, 7 and 14 after PQ injection. The b-wave ratio was defined as b-wave amplitude of right eye/ b-wave amplitude of left eye at definite time point. The fold of b-wave ratio represented the b-wave ratio at day 3, 7 or 14 / b-wave ratio at day 0. The results represented six rats for each group in each time point. (*P < 0.05 compared with PQ only or PBS-treated group; # *P* < 0.05 compared with normal; & *P*< 0.05 by two-way ANOVA with post hoc Bonferroni test).

The b-wave ratio of the ERG was greatly reduced to about 0.5 at day 3 after injection of 0.5 mM PQ. Treatment with low-dose or high-dose of COS significantly enhanced the recovery of the b-wave ratio at day 3 (*P*<0.05, low-dose vs. PQ only or PBS-treated group ; *P*<0.01, high-dose vs. PQ only or PBS-treated group; two-way ANOVA with post hoc Bonferroni test; N=6 in each group), 7 (*P*< 0.01, low-dose vs. PQ only or PBS-treated group; *P*<0.001, high-dose vs. PQ only or PBS-treated group; two-way ANOVA with post hoc Bonferroni test ; N=6 in each group), and 14 (*P*<0.001, low-dose vs. PQ only or PBS-treated group; *P*<0.001, high-dose vs. PQ only or PBS-treated group; two-way ANOVA with post hoc Bonferroni test ; N=6 in each group). The b-wave ratio was significantly higher in those treatment with high-dose than low-dose COS, both at day 3 (*P*= 0.02; two-way ANOVA with post hoc Bonferroni test; N=6 in each group), day 7 (*P*= 0.008; two-way ANOVA with post hoc Bonferroni test; N=6 in each group) and day 14 (*P*=0.02; two-way ANOVA with post hoc Bonferroni test; N=6 in each group). At day 14, the b-wave ratio was restored to about 0.85 in the high-dose COS group (Figure 1).

### Effects of COS on retinal morphology and retinal cell counting

**Figure 2:**
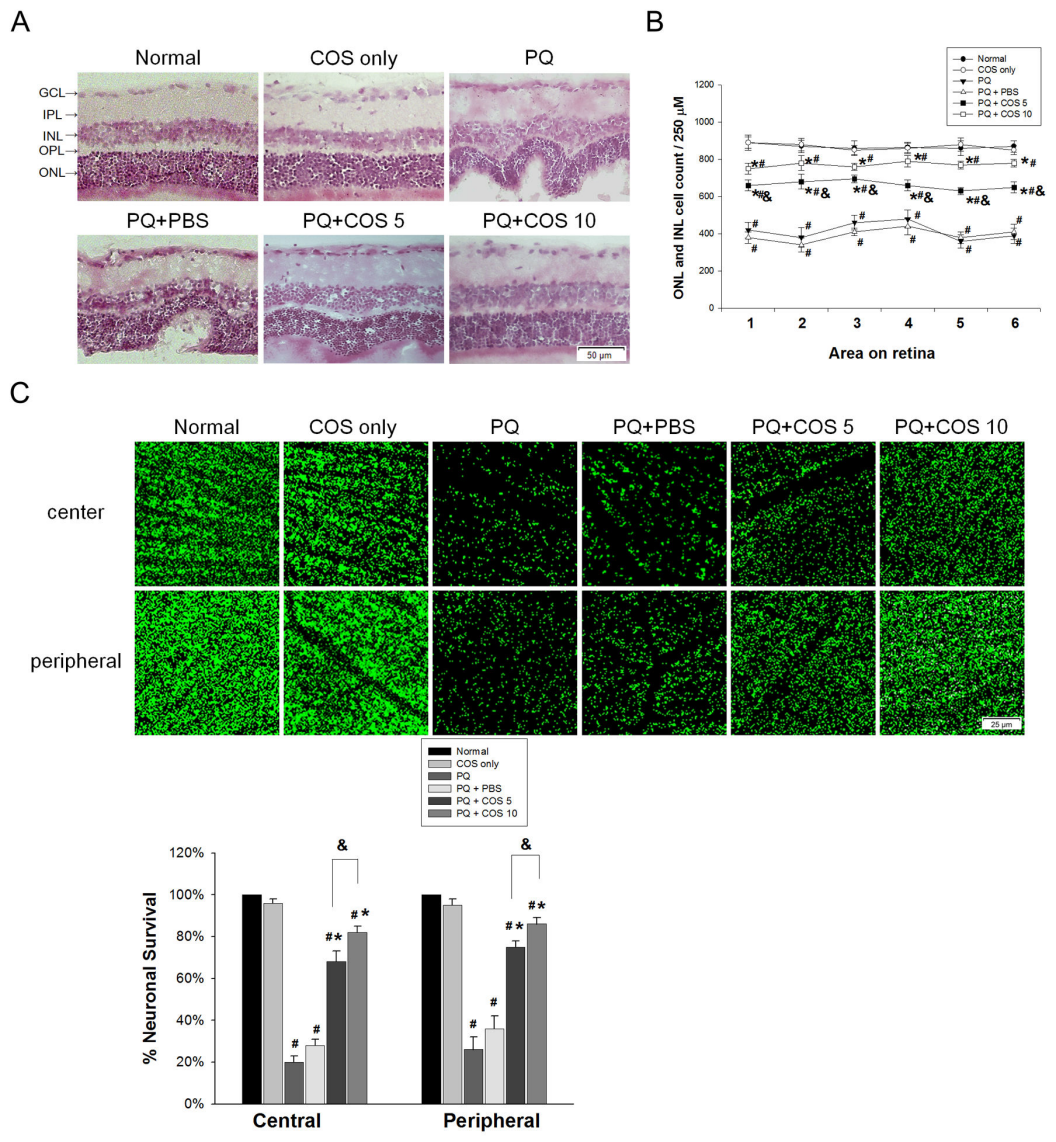
Effects of COS on retinal morphology, retinal cells and ganglion cell layer count. (A). PQ-injected rats showed diffuse thinning of inner and outer nuclear layer (INL, ONL) and folds in ONL, while low-dose or high-dose COS treatment showed better preservation of the INL and ONL at day 14. (B). low-dose and high-dose COS-treated group significantly preserved more cells in INL and ONL than PBS-treated and untreated group. (N=5 in each group). (C). Representative confocal images of Neu N-labeled neurons (green) in the regions of center and peripheral flat-mounted retinas of four groups. Neu N is the neuron marker for the quantification of surviving neurons in the ganglion cell layer (GCL). The percentage of surviving neurons in the GCL was significantly higher in COS-treated group than PBS-treated and untreated groups both in the center and peripheral regions of retinas at day 14. (N=3 in each group). (*P < 0.05 compared with PQ only or PBS-treated group; # *P* < 0.05 compared with normal; & *P*< 0.05 by ANOVA test with post hoc Bonferroni test).

The most striking findings in the retinas of PQ only and PBS-treated group are folds in the outer nuclear layer (ONL) and diffuse thinning, vacuolization in the inner and outer nuclear layers. Treatment with low-dose or high-dose COS showed better preservation of the INL and ONL, with fewer folds in the ONL at day 14. After counting the cells in INL and ONL, the number of cells was significantly higher in the low-dose and high-dose COS treatment groups than in the PQ or PBS-treated groups (*P*<0.05, low-dose vs. PQ only or PBS-treated group; *P*<0.01, high-dose vs. PQ only or PBS-treated group; one-way ANOVA with post hoc Bonferroni test; N=5 in each group) (Figure 2A and B).

### Effects of COS on survival of retinal neurons

Whole retina flat mounts were stained for the neuronal marker Neu N to quantify the number of surviving neurons in the ganglion cell layer (GCL). We found intravitreal injection of PQ induced a diffuse loss of retinal neurons in the GCL. Low-dose or high-dose COS treatment lead to significantly higher survival of Neu N-postive neurons in the GCL, when compared with the PQ only or PBS-treated groups at day 14 (*P* < 0.001, low-dose vs. PQ only or PBS-treated group; *P* < 0.001, high-dose vs. PQ only or PBS-treated group; one-way ANOVA with post hoc Bonferroni test;; N=3 in each group). The effects of decreased neuron loss in ganglion cell layer were more noticeable in the high-dose COS treatment group than in the low-dose group (*P*= 0.01, central region; *P*=0.03, peripheral region; one-way ANOVA with post hoc Bonferroni test; N=3 in each group) (Figure 2C and D).

### Effects of COS on retinal apoptosis

**Figure 3:**
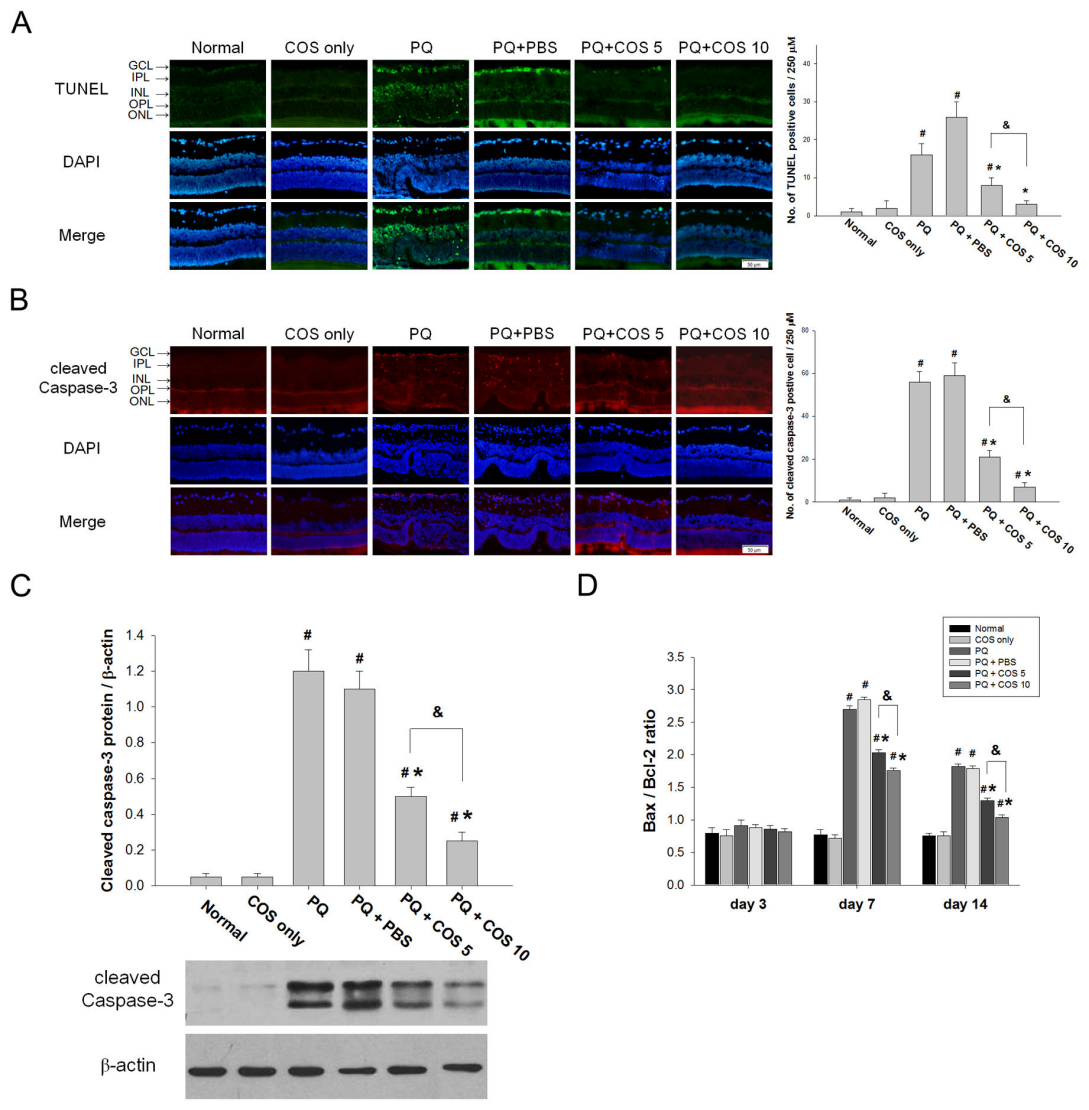
Effects of COS on retinal cell apoptosis. (A). Retinal apoptosis was evaluated by using TUNEL stain. In PQ only and PBS-treated group, TUNEL-positive cells were detected in GCL of the retina, whereas only sparse TUNEL-positive cells in GCL of retina were noted in COS-treated group at day 14. (B). Retinal sections and staining with anti-cleaved caspase-3 antibody. There are significantly few cleaved caspase-3-positive cells in GCL of retina in COS-treated group than in the PQ only and PBS-treated group at day 14. (C).Effects of COS on the expression of cleaved caspase-3 protein in retina. The protein level of cleaved caspase-3 in retina was evaluated by western blot analysis. The cleaved caspase-3 protein was significantly lower in the low-dose and high-dose COS treatment group than in the PQ only or PBS-treated group at day 14. (D). Effects of COS on Bax/Bcl-2 ratio. The amounts of Bax and Bcl-2 in retina were measured by ELISA and the ratio was calculated. Treatment with low-dose or high-dose of COS caused significantly decrease in Bax/Bcl-2 ratio at day 7 and 14. (*P < 0.05 compared with PQ only or PBS-treated group; # *P* < 0.05 compared with normal; & *P*< 0.05 by ANOVA test with post hoc Bonferroni test; N=3 for each group in each time point).

At day 14, more TUNEL-positive cells in the GCL of the retina were noted the in the PQ only and PBS-treated groups, while only few TUNEL-positive cells were noted in the low-dose COS treatment group and nearly no TUNEL-positive cells were detected in the high-dose COS treatment group. After counting the number of apoptotic cells in the retina, there were statistically fewer apoptotic cells in the low-dose and high-dose COS treatment group, than in the PQ only or PBS-treated group (*P*< 0.01, low-dose vs. PQ only or PBS-treated group; *P*<0.001, high-dose vs. PQ only or PBS-treated group; one-way ANOVA with post hoc Bonferroni test; N=3 in each group) (Figure 3A).

Similarly, more cleaved caspase-3-positive cells in GCL and INL of the retina were noted the in the PQ only and PBS-treated group, whereas only sparse cleaved caspase-3-positive cells were found in the low-dose and high-dose COS treatment groups at day 14. Comparisons of the PQ only or PBS-treated group, low-dose, and high-dose COS treatment groups had significantly fewer cleaved caspase-3-positive cells in the retina at 14 days (*P*<0.001, low-dose vs. PQ only or PBS-treated group; *P*<0.001, high-dose vs. PQ only or PBS-treated group; one-way ANOVA with post hoc Bonferroni test; N=3 in each group) (Figure 3B). Consistent with the results obtained from fluorescent immunohistochemistry, Western blot analysis showed the protein level of cleaved caspase-3 was significantly lower in the low-dose and high-dose COS treatment group than in the PQ only or PBS-treated group at day 14 (*P*<0.001, low-dose vs. PQ only or PBS-treated group; *P*<0.001, high-dose vs. PQ only or PBS-treated group; one-way ANOVA with post hoc Bonferroni test; N=3 in each group). In addition, the level of cleaved caspase-3 was more reduced in the high-dose COS treatment group than in the low-dose COS treatment group (*P*<0.01; one-way ANOVA with post hoc Bonferroni test; N=3 in each group) (Figure 3C). 

### Effects of COS on Bax/Bcl-2 ratio

The Bax/Bcl-2 ratio was significantly higher in the PQ only and PBS-treated group than in the normal group at day 7 (*P*<0.001, PQ only vs. normal; *P*<0.001, PBS-treated group vs. normal; two-way ANOVA with post hoc Bonferroni test; N=3 in each group) and 14 (*P*<0.01, PQ only vs. normal; *P*<0.01, PBS-treated group vs. normal; two-way ANOVA with post hoc Bonferroni test; N=3 in each group). The low-dose and high-dose COS treatment groups significantly reversed the increase in the Bax/Bcl-2 ratio, when compared with the PQ only or PBS-treated group at day 7 (*P*<0.01, low-dose vs. PQ only or PBS-treated group; *P*<0.01, high-dose vs. PQ only or PBS-treated group; two-way ANOVA with post hoc Bonferroni test ; N=3 in each group) and 14 (*P*<0.01, low-dose vs. PQ only or PBS-treated group; *P*<0.01, high-dose vs. PQ only or PBS-treated group; two-way ANOVA with post hoc Bonferroni test ; N=3 in each group) (Figure 3D).

### Effects of COS on retinal reactive oxygen species

**Figure 4:**
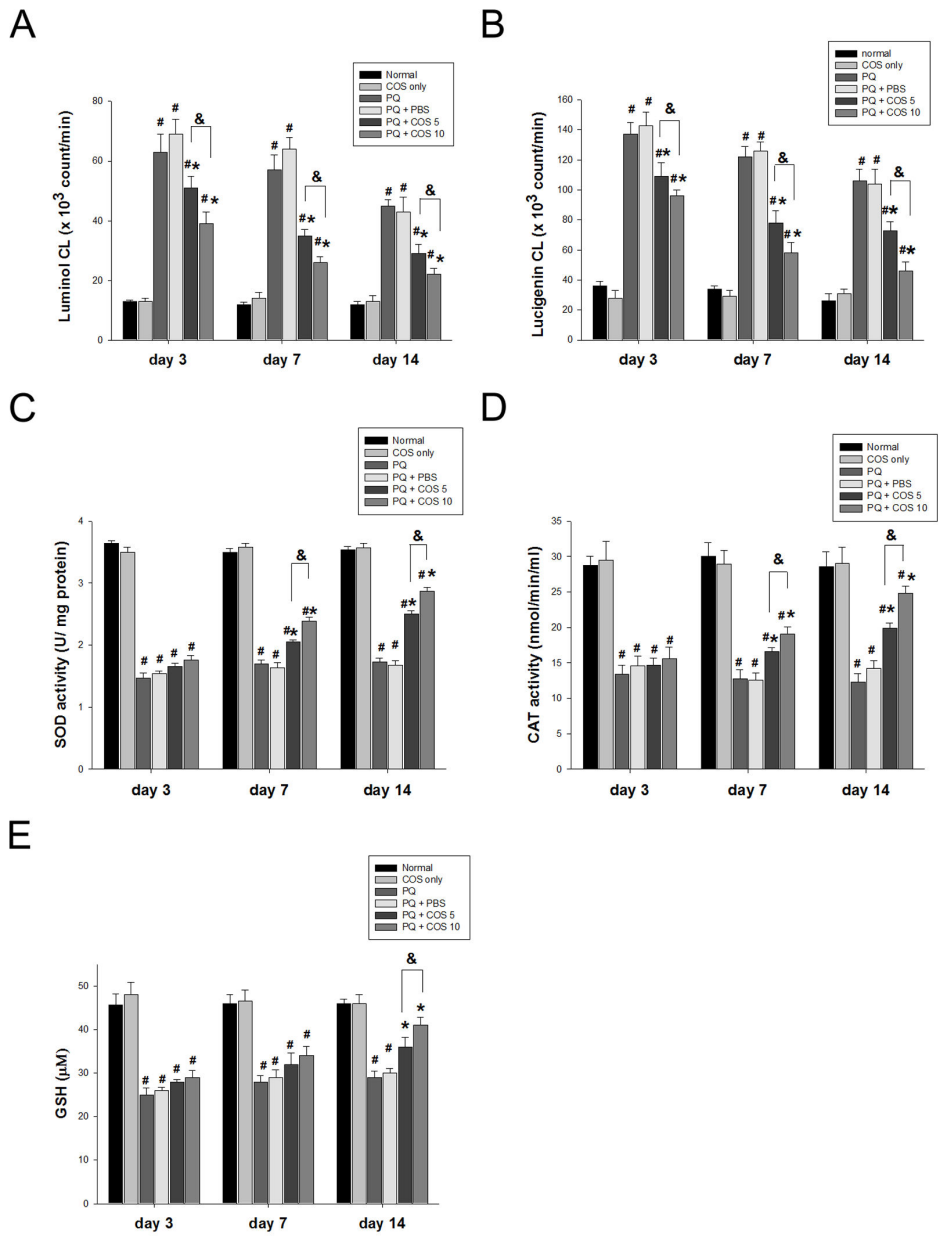
Effects of COS on the amounts of free radicals, antioxidant enzyme activity and glutathione levels. The amounts of free radicals in retina were assessed by using (A) luminol- and (B) lucigenin- dependent chemiluminenscences. The activities of anti-oxidative enzyme: (C) superoxide dismutase (SOD) and (D) catalase and (E) glutathione (GSH) levels in retinas at indicated time points were evaluated by using ELISA. (*P < 0.05 compared with PQ only or PBS-treated group; # *P* < 0.05 compared with normal; & *P*< 0.05 by two-way ANOVA with post hoc Bonferroni test; N=3 for each group in each time point).

We examined the generation of reactive oxygen species in retinas by lucigenin- and luminol-enhanced CL. Luminol- and lucigenin-enhanced CL predominantly detect reactive oxygen species (ROS) and superoxide. The mean luminal-enhanced CL count showed significant reductions in the low-dose and high-dose COS treatment group, when compared with that in the PQ only or PBS-treated group at days 3 (*P*<0.01, low-dose vs. PQ only or PBS-treated group; *P*<0.001, high-dose vs. PQ only or PBS-treated group; two-way ANOVA with post hoc Bonferroni test; N=3 in each group), 7 (*P*<0.001, low-dose vs. PQ only or PBS-treated group; *P*<0.001, high-dose vs. PQ only or PBS-treated group; two-way ANOVA with post hoc Bonferroni test; N=3 in each group) and 14 (*P*<0.01, low-dose vs. PQ only or PBS-treated group; *P*<0.001, high-dose vs. PQ only or PBS-treated group; two-way ANOVA with post hoc Bonferroni test; N=3 in each group) (Figure 4A). Similarity, the low-dose and high-dose COS treatment groups experienced more significant diminished elevations in lucigenin-enhanced CL count, than did the PQ only and PBS-treated groups at day 3 (*P*<0.01, low-dose vs. PQ only or PBS-treated group; *P*<0.001, high-dose vs. PQ only or PBS-treated group; two-way ANOVA with post hoc Bonferroni test ; N=3 in each group), 7 (*P*<0.001, low-dose vs. PQ only or PBS-treated group; *P*<0.001, high-dose vs. PQ only or PBS-treated group; two-way ANOVA with post hoc Bonferroni test; N=3 in each group) and 14 (*P*<0.01, low-dose vs. PQ only or PBS-treated group; *P*<0.001, high-dose vs. PQ only or PBS-treated group; two-way ANOVA with post hoc Bonferroni test; N=3 in each group). These effects were more remarkable in the high-dose than the low-dose COS treatment group (*P*=0.03, day 3; *P*=0.01 day 7; *P*=0.004 day 14; two-way ANOVA with post hoc Bonferroni test ; N=3 in each group) (Figure 4B). 

### COS treatment increased activity of the antioxidant enzymes

SOD and catalase are the most important enzymes of the antioxidant defense system. As shown in Figure 4 C and D, intravitreal injection of PQ resulted in decreased activity of the SOD and catalase enzymes by about 3.2- and 2.1-fold, respectively, compared to normal. Treatment with low-dose and high-dose COS significantly attenuated the decrease of SOD activity at days 7 (*P*<0.05, low-dose vs. PQ only or PBS-treated group; *P*<0.01, high-dose vs. PQ only or PBS-treated group; two-way ANOVA with post hoc Bonferroni test; N=3 in each group )and 14 (*P*<0.01, low-dose vs. PQ only or PBS-treated group; *P*<0.001, high-dose vs. PQ only or PBS-treated group; two-way ANOVA with post hoc Bonferroni test; N=3 in each group) in a dose-dependent manner (*P* < 0.05, low-dose vs. high-dose both at day 7 and 14; two-way ANOVA with post hoc Bonferroni test; N=3 in each group). Similarly, PQ-induced decreases in catalase activity were significantly inhibited by low-dose and high-dose COS at day 7 (*P*<0.05, low-dose vs. PQ only or PBS-treated group; *P*<0.01, high-dose vs. PQ only or PBS-treated group; two-way ANOVA with post hoc Bonferroni test; N=3 in each group) and 14 (*P*<0.01, low-dose vs. PQ only or PBS-treated group; *P*<0.001, high-dose vs. PQ only or PBS-treated group; two-way ANOVA with post hoc Bonferroni test; N=3 in each group) in a dose-dependent manner (*P* < 0.05, low-dose vs. high-dose at day 7; *P* < 0.01, low-dose vs. high-dose at day 14; two-way ANOVA with post hoc Bonferroni test; N=3 in each group). 

Treatment with COS resulted in significant increases in GSH levels in a dose-dependent manner (*P* < 0.05, low-dose vs. high-dose; two-way ANOVA with post hoc Bonferroni test; N=3 in each group), compared with the PQ only or PBS-treated groups at day 14 (*P*<0.05, low-dose vs. PQ only or PBS-treated group; *P*<0.01, high-dose vs. PQ only or PBS-treated group; two-way ANOVA with post hoc Bonferroni test; N=3 in each group) (Figure 4E).

### Effects of COS treatment on retinal oxidative damages

**Figure 5:**
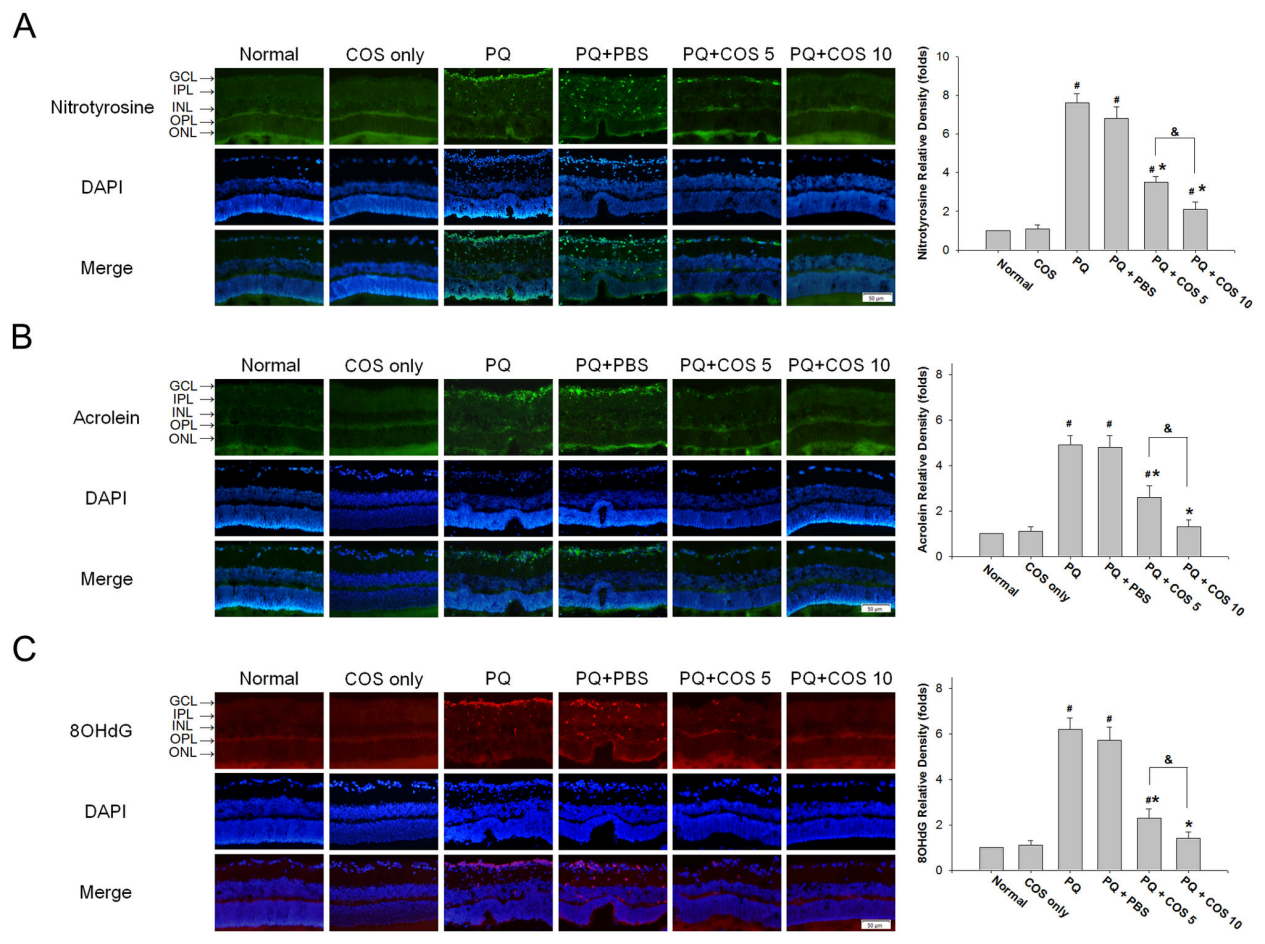
Effects of COS treatment on oxidative damages to retina. Retinal oxidative damages were evaluated by immunohistochemical staining of (A) nitrotysine (B) acrolein, and (C) 8-hydroxy-2'-deoxyguanosine (8-OHdG) at day 14. For quantitation of immunostaining, we first determined the immunostaining index, which could be measured and calculated from the following formula: Σ [(immunostaining density-threshold) × area (pixels)] / total cell number. The relative density of immunostaining was defined as immunostaining index of PQ only, COS only, PBS-treated, low-dose or high-dose COS treated groups / immunostaining index of normal group. Treatment with COS decreased the staining for nitrotysine acrolein and 8-OHdG in the retinas at day 14. (*P < 0.05 compared with PQ only or PBS-treated group; # *P* < 0.05 compared with normal; & *P*< 0.05 by ANOVA with post hoc Bonferroni test; N=3 in each group).

High levels of ROS generation can result in oxidative tissue damage. We tested retinal oxidative damage at day 14 after PQ injection to protein, lipid, and DNA by staining retinal sections with nitrotyrosine, acrolein, and 8-OHdG. As shown in Figure 5, intravitreal PQ injection caused a striking increase in immunohistochemical staining for acrolein, nitrotyrosine, and 8-OHdG in the retina. At day 14, the low-dose and high-dose COS treatment groups showed significantly decreased the relative density of nitrotyrosine, acrolein and 8-OHdG in the retinas in a dose-dependent manner (*P*<0.05 for nitrotyrosine, acrolein and 8-OHdG, low-dose vs. high-dose; one-way ANOVA with post hoc Bonferroni test; N=3 in each group), when compared with the PQ only or PBS-treated groups (*P*<0.01, low-dose vs. PQ only or PBS-treated group; *P*<0.001, high-dose vs. PQ only or PBS-treated group; two-way ANOVA with post hoc Bonferroni test; N=3 in each group).

### Effects of COS on PQ-stimulated RGC-5 cells viability, apoptosis, and anti-oxidative enzymes

**Figure 6:**
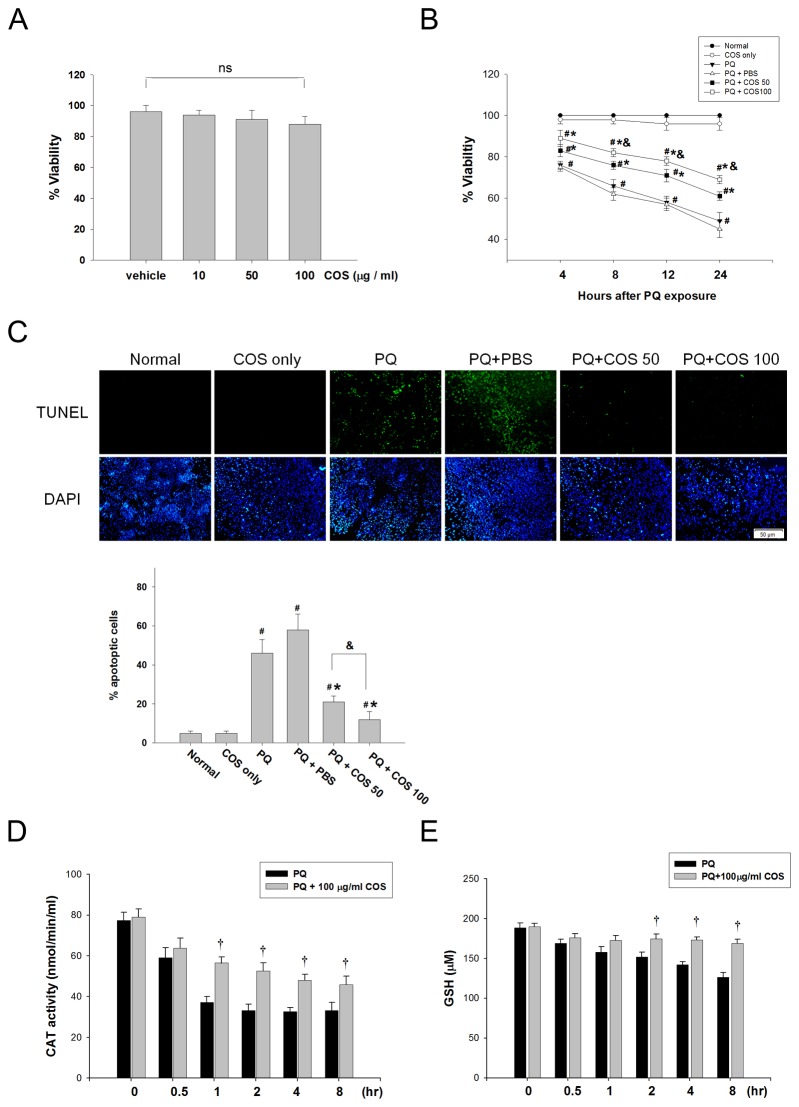
The influence of COS on PQ-induced cell death, apoptosis and antioxidative enzyme in RGC-5 cells. (A) Effects of COS on RGC-5 viability determined by MTT assays. RGC-5 cell were incubated for 24 h with vehicle or COS at indicated concentrations. (B) COS decreased PQ-stimulated RGC-5 cell death. RGC-5 cell were stimulated with 0.5 mM PQ and treated with 50 or 100 μg/ml COS for indicated time. (C) Inhibition by COS of PQ-induced apoptosis. RGC-5 cell were stimulated with 0.5 mM PQ and treated with 50 or 100 μg/ml COS or PBS for 6 hours. Cells apoptosis was analyzed by TUNEL stain. Percent apoptotic RGC-5 cells were obtained by counting at least 400 cells. Data were expressed as mean ± SD. ns, Non-significant compared with vehicle-treated control. (*P < 0.05 compared with PQ only or PBS-treated group; # *P* < 0.05 compared with normal; & *P*< 0.05 by ANOVA with post hoc Bonferroni test; N=3 in each group) (D) COS activated catalase activity in PQ-stimulated RGC-5. RGC-5 cell were stimulated with 0.5 mM PQ and treated with 100 μg/ml COS for indicated time. (E) COS increased GSH levels in PQ-stimulated RGC-5. (†*P* < 0.05 compared with PQ only by Student’s t-test; N=3 in each group).

We first examined the toxicity of COS on RGC-5 cells by the MTT assay. We found that COS, at concentrations ranging from 10 to 100 µg/ml, had no effect on RGC-5 cell viability (*P*=0.31; one-way ANOVA, N=3) (Figure 6A). Subsequently, we examined the effects of COS on the viability of PQ-stimulated RGC-5 cells. Cell viability was significantly reduced at 4, 8, 12, and 24 hr after exposure to 0.5 mM PQ, when compared with normal control (*P*<0.01 for 4 hr; *P*<0.001 for 8, 12 and 24 hr; two-way ANOVA with post hoc Bonferroni test, N=3 in each group). Treatment with COS increased cell viability in PQ-stimulated cells in a dose-dependent manner (P<0.05 for 8, 12 and 24 hr, low-dose vs. high dose; two-way ANOVA with post hoc Bonferroni test; N=3 in each group). The protective effect of 50 and 100 µg/ml COS reached statistical significance at 4, 8, 12, and 24 hr after treatment, when compared with the PQ only or PBS-treated group (*P*<0.05 for 4 hr, P<0.01 for 8, 12, and 24 hr, low-dose vs. PQ only or PBS-treated group; *P*<0.05 for 4 hr, P<0.01 for 8, 12, and 24 hr, high-dose vs. PQ only or PBS-treated group; two-way ANOVA with post hoc Bonferroni test; N=3 in each group )(Figure 6B).

The effect of COS on PQ-induced apoptosis in RGC-5 cells was examined by TUNEL staining. Treatment with COS at concentration of 50 and 100 µg/ml markedly reduced the PQ-induced RGC-5 cell apoptosis (*P*<0.01, low-dose vs. PQ only or PBS-treated group; *P*<0.001, high-dose vs. PQ only or PBS-treated group; one-way ANOVA with post hoc Bonferroni test; N=3 in each group) (Figure 6C).

Next, we examined the effect of COS on the anti-oxidative enzyme catalase activity and GSH levels in PQ-stimulated RGC-5 cells. COS, at the concentration of 100 µg/ml, caused a marked increase in catalase activity at 1, 2, 4 and 8 hr after treatment (*P*<0.01 for 1, 2, 4 and 8 hr, COS vs. PQ only; Student’s t-test; N=3 in each group) and in GSH levels at 2, 4 and 8 hours after treatment, compared to PQ-treated cells (*P*<0.05 for 2 hr, *P*<0.01 for 4 and 8 hr, COS vs. PQ only; Student’s t-test; N=3 in each group)(Figure 6D and E).

### Influence of COS on the activation of NF-κB in retina and RGC-5 cells in vitro

**Figure 7:**
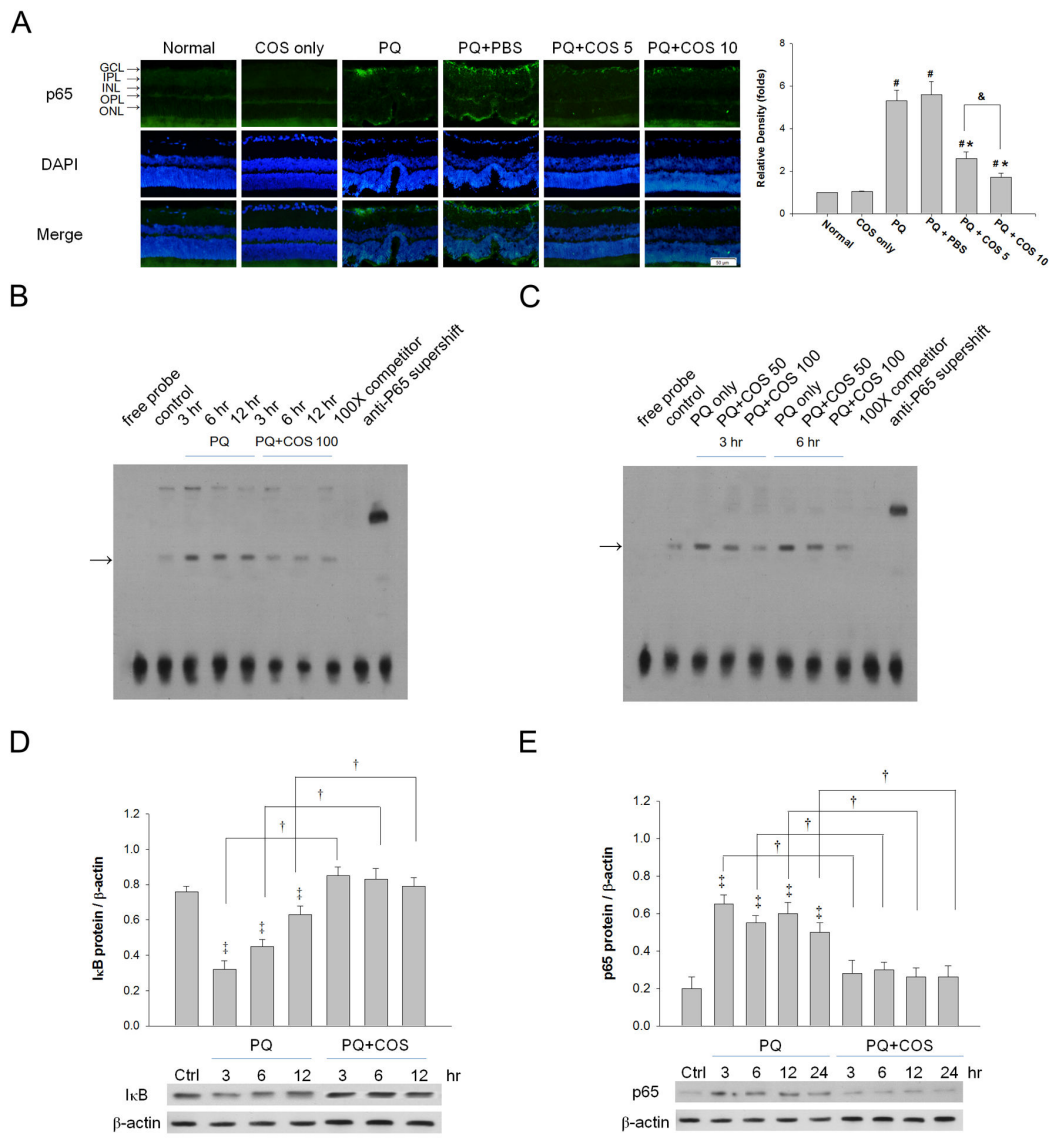
Influence of COS on the activation of NF-κB in retina and RGC-5 cells. (A)Immunohistochemical study of the expression of the NF-κB p65 subunit in retinas. For quantitative immunohistochemistry for NF-κB p65, we first determined the p65 index, which could be measured and calculated from the following formula: Σ [(p65 staining density-threshold) × area (pixels)] / total cell number. The relative density of p65 was defined as p65 index of PQ only, COS only, PBS-treated, low-dose or high-dose COS treated groups / p65 index of normal group. Data were expressed as mean ± SD. (*P < 0.05 compared with PQ only or PBS-treated group; # *P* < 0.05 compared with normal; & *P*< 0.05 by ANOVA with post hoc Bonferroni test; N=3 in each group). (B) COS decreased the amount of activated NF-κB translocated into nucleus in PQ-stimulated RGC-5 in a time-dependent and (C) dose-dependent manner. RGC-5 cell were stimulated with 0.5 mM PQ and treated with 50 or 100 μg/ml COS or PBS for indicated time. The amount of activated NF-κB translocated into nucleus in PQ-stimulated RGC-5 was measured by using EMSA. (D) Evaluation of the sequential change of IκB with Western blot analysis. The Y scale represents the ratio of IκB blot density to the β-actin blot density. (E) Evaluation of the sequential change of NF-κB p65 with Western blot analysis. The level of NF-κB p65 significantly reduced in RGC-5 cells after 3 hours of PQ stimulation. The Y scale represents the ratio of NF-κB p65 blot density to the β-actin blot density. Data were expressed as mean ± SD. (†*P* < 0.05 compared with PQ; ‡*P* < 0.05 compared with normal control by Student’s t-test; N=3 in each group).

Immunohistochemical studies showed that the expression of the p65 subunit of NF-κB, which is available for specific antibody binding only after its dissociation from IκB, was elevated in retinas after PQ stimulation. Treatment with low- and high-dose COS greatly reduced the expression of p65 in retina, indicating that the activation of NF-κB was suppressed. The relative density of p65 was significantly reduced in the high-dose treatment group than in the low-dose group (*P*<0.05; low-dose vs. high-dose; one-way ANOVA with post hoc Bonferroni test; N=3 in each group) (Figure 7A).

We found that treatment with 100 μg/ml COS for 3, 6, and 12 hours decreased the amount of activated NF-κB translocated into nucleus in PQ-stimulated RGC-5 cells (Figure 7B). Moreover, COS treatment for 3 and 6 hours resulted in decreased the amount of activated NF-κB translocated into nucleus in a dose-dependent manner. The increased activation of NF-κB in RGC-5 cells after PQ stimulation was inhibited markedly by treatment with 100 μg/ml COS (Figure 7C). Adding a 100-fold molar excess of unlabelled NF-κB probe completely inhibited the binding of the labeled probe to the NF-κB/ DNA complex.

The levels of IκB were significantly reduced in RGC-5 cells after 3 hours of PQ stimulation. Treatment with 100 μg/ml COS significantly increased the expression of IκB at 3, 6, and 12 hours, when compared with untreated PQ-stimulated cells (*P*<0.01 for 3 and 6 hr, *P*<0.05 for 12 hr; time-matched comparison COS vs. PQ; Student’s t-test; N=3 in each group). There was no significant change in IκB levels in cells treated with 100 μg/ml COS at 3, 6, and 12 hours, compared to normal cells (*P* >0.05 for 3, 6 and 12 hr; COS vs. normal; one-way ANOVA with post hoc Bonferroni test; N=3 in each group)(Figure 7D).

Determination of the level of NF-κB p65 at various time points showed that p65 increased significantly after 3 hours of PQ stimulation, and the cells treated with 100 μg/ml COS caused significantly decreases in p65 levels at 3, 6, 12 and 24 hours compared with untreated PQ-stimulated cells (*P*<0.05 for 3, 6, 12 and 24 hr; time-matched comparison COS vs. PQ; Student’s t-test; N=3 in each group). There was no significant change in p65 levels in cells treated with 100 μg/ml COS at 3, 6, 12 and 24 hours, compared to those in normal cells (*P*>0.05 for 3, 6, 12 and 24 hr; time-matched comparison COS vs. normal; one-way ANOVA with post hoc Bonferroni test; N=3 in each group)(Figure 7E).

### Effects of COS on the expression of iNOS and MCP-1 mRNA in RGC-5 cells in vitro

**Figure 8:**
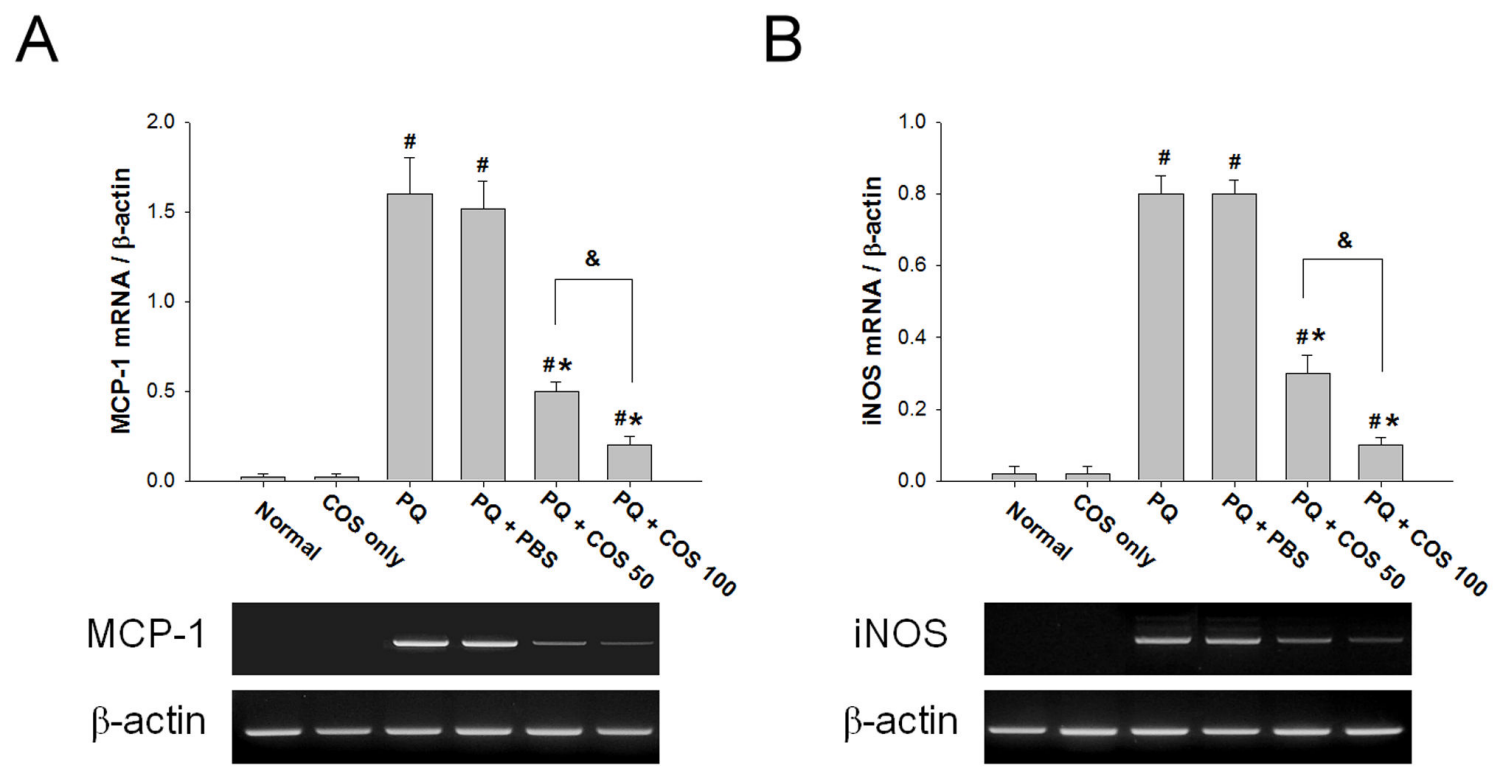
Effects of COS on the expression of MCP-1 and iNOS mRNA in RGC-5 cells. RGC-5 cells were stimulated with 0.5mM PQ and then treated with 50 μg/ml (low-dose) or 100 μg/ml (high-dose) COS for 12 hours. The mRNA expression levels of MCP-1 (A) and iNOS (B) were evaluated by semi-quantitative RT-PCR. The expression of MCP-1 and iNOS mRNA was significantly decreased in the low-dose and high-dose COS group than in the PQ only or PBS-treated group. In addition, the levels of iNOS and MCP-1 mRNA were more reduced in the high-dose than in the low-dose COS group. Data were expressed as mean ± SD. (*P < 0.05 compared with PQ only or PBS-treated group; # *P* < 0.05 compared with normal; & *P*< 0.05 by ANOVA test with post hoc Bonferroni test; N=3 in each group).

Because COS effectively reduced the activation of NF-κB, we further determined whether COS could inhibit the expression of NF-κB-dependent genes iNOS and MCP-1 in RGC-5 cells. As shown in Figure 8, the mRNA expression level of iNOS and MCP-1 was significantly lower in low-dose and high-dose COS treatment group than in the PQ only or PBS-treated group (P<0.01 for both iNOS and MCP-1; low-dose vs. PQ only or PBS-treated group; *P*<0.001 for both iNOS and MCP-1, high-dose vs. PQ only or PBS-treated group; N=3 in each group). In addition, the levels of iNOS and MCP-1 mRNA were more reduced in the high-dose than in the low-dose COS treatment group (P<0.01 for both iNOS and MCP-1, low-dose vs. high-dose; one-way ANOVA with post hoc Bonferroni test; N=3 in each group). 

## Discussion

COS has been recommended as a superior agent to other antioxidants, because of its biocompatibility, biodegradability, non-toxicity, and absorption properties [18,19]. In addition, COS is a natural product, abundant in marine foods, that aides in the prevention or treatment of disease. In this study, we found that COS enhanced restoration of retinal function and reversal of retinal damage in a dose-dependent manner. This was demonstrated in a rat model of oxidative-induced retinal disease by the recovery of ERG b-wave amplitude and the preservation of retinal morphology and neurons in retinal ganglion layer. Furthermore, COS treatment increased the activity of antioxidant enzymes: SOD and catalase, decreased ROS levels in retina and thus attenuated oxidative retinal damage, which may have contributed to the protection of the retinal cells from apoptosis. Importantly, we showed COS treatment reduced the expression of NF-κB p65 in the retina. The in vitro study demonstrated the dose-dependent anti-oxidative and protective effects of COS in PQ-stimulated RGC-5 cells, consistent with the results obtained in vivo. Treatment with COS increased IκB expression, meanwhile, decreased p65 expression, and thus reduced DNA-binding activity of NF-κB and NF-κB-dependent genes MCP-1 and iNOS expression in PQ-stimulated RGC-5 cells. Taken together, these results suggest that COS may be effective for the treatment of oxidative-induced retinal diseases through inhibition of NF-κB signaling and apoptosis of retinal cells.

It is well known that excess production of ROS results in continuous and accumulative oxidative damage to cellular components, alters many cellular functions, and leads to apoptotic cell death [20,21]. In this study, we evaluated the generation of ROS in the retina by using the luminol and lucigenin CL assay. The luminol detects H_2_O_2_, OH^−^, hypochlorite, peroxynitrite, and lipid peroxyl radicals, whereas lucigenin is particularly sensitive to superoxide radical [22]. We found intravitreous PQ injection significantly increase luminol- and lucigenin-CL levels in the retina, whereas COS treatment decreased both the luminol- and lucigenin-CL levels. Moreover, acrolein, nitrotyrosine and 8-OHdG are generated by oxidative damage to lipids, proteins, and DNA, respectively, and are widely used as biomarkers of oxidative stress [23]. In accordance with the reduced ROS production, COS treatment effectively attenuated retinal staining for acrolein, nitrotyrosine, and 8-OHdG, indicating COS treatment ameliorated damages to lipids, proteins, and DNA of retinal cells and thus reduced the number of apoptotic cells, enhancing the survival of retina cells.

Bax is a proapoptotic protein that may translocate to and permeabilize the mitochondria after apoptotic stimuli, causing release of cytochrome c, and ultimately leading to an apoptotic cascade [24,25]. Bcl-2 can counter the actions of Bax and thereby prevent the cell from succumbing to apoptosis [26]. Thus, the Bax/Bcl-2 ratio plays an important role in determining the cell’s fate (i.e., survival or death) following an apoptotic stimulus. In our study, low- and high-doses of COS effectively reduced the number of TUNEL-positive and caspase 3-positive apoptotic cells in retinas, with a concomitant decrease in the Bax/Bcl ratios at day 7 and 14. These results indicated that COS ameliorated PQ-induced retinal degeneration by inhibiting apoptotic pathways.

Excess ROS produced in the body are scavenged by antioxidant enzymes, which are important defense mechanisms in controlling the levels of ROS and preventing oxidative cell death [27,28]. SOD and catalase, responsible for the detoxication of O_2_^-^ and H_2_O_2_ respectively, are the most important antioxidative enzymes [29]. Moreover, GSH plays a critical role in regulating the intracellular redox system to defend cells from oxidative stress [30]. GSH directly reacts with ROS and functions as a cofactor of glutathione peroxidase. In the present study, significant decreases in SOD, catalase, and GSH were observed in eyes after exposure to PQ, indicating impairment in antioxidant defenses. Nonetheless, COS treatment increased SOD and catalase activity and GSH levels. This was accompanied by a decrease in ROS production and cellular damages. These results suggest that enhancement of endogenous antioxidant preservation may represent another protective mechanism of COS treatment. 

In this study, we showed that the activities of anti-oxidative enzymes SOD and catalase were upregulated at day 7, whereas the production of ROS began to decline at day 3. This discrepancy implied that the ROS-lowing effects with COS treatment were not wholly attributed to the activation of anti-oxidative enzymes. Several studies demonstrated that COS is an antioxidant with free radical scavenging ability [31,32]. Je et al. have shown that COS could quench various radicals by the action of nitrogen on C-2 position of COS [31]. Xie et al, reported that the scavenging mechanisms of chitosans are related to their hydrogen donating ability to free radicals to form stable molecules [32]. 

NF-κB is a redox-sensitive transcription factor, and has been implicated in the cellular response to oxidative stress [33,34]. The roles of COS on NF-κB activation are controversial. Most studies have demonstrated that COS inhibited NF-κB activation and thus suppressed NF-κB-dependent genes [35-37]. Yousef et al, demonstrated that COS markedly attenuated NF-κB activation and the production of TNF-α and IL-6 in human colonic epithelial cell-like T84 cells [38]. In contrast, few reports showed that oligochitosan can activate NF-κB [39]. In the current study, we found that COS treatment reduced p65 subunit expression in retina as demonstrated by immunohistochemical staining. Our in vitro study with RGC-5 cells found that treatment with COS increased the expression of IκB and attenuated the increase of p65 after PQ-stimulation. Taken together, these results clearly indicated that COS could inhibit the activation of NF-κB in PQ-induced retinal damage in vivo and in vitro. In addition, it is well known that ROS is a potent activator of NF-κB. Several studies showed that the inhibitory ability of some antioxidants on NF-κB activation is through a decrease in the amount of ROS [40,41]. Our results showed that COS decreased ROS production and caused a substantial induction of GSH, implying that COS inhibited NF-κB activation in our study may partly be mediated by reducing the amount of ROS in the retina.

## Materials and Methods

### Reagents

Chitosan oligosaccharide and paraquat were purchased from Sigma-Aldrich (St. Louis, MO, USA). The DNA fragmentation detection kit (TUNEL) was obtained from Calbiochem (La Jolla, CA, USA). Green Fluorescent Protein (GFP) antibody was purchased from BioVision (Mountain View, CA, USA). Mounting Medium with 4', 6-diamidino-2-phenylindole (DAPI) and phycoerythrin Streptavidin antibody were obtained from Vector Laboratories (Burlingame, CA, USA). Anti-nitrotyrosine was obtained from Abcam (Cambridge, MA, USA), anti-8-Hydroxy-2'-deoxyguano-sine from JaICA (Fukuroi, Shizuoka, Japan) and anti-conjugated acrolein antibody from Advanced Targeting Systems (San Diego, CA, USA). Anti-p65 antibody was from Rockland (Gilbertsville, PA, USA). Anit- cleaved caspase-3 antibody was purchased from Cell Signaling (Denvers, MA, USA)

### Animals and retinal oxidative stress animal model

Sprague-Dawley (SD) rats weighting 150 to 200 g were used in all experiments. This study was carried out in strict accordance with the recommendations in the Guide for the Care and Use of Laboratory Animals of the National Institutes of Health. The protocol was approved by the Committee on the Ethics of Animal Experiments of the National Taiwan University Hospital (Permit Number: 2009-0383). All surgery was performed under sodium pentobarbital anesthesia, and all efforts were made to minimize suffering. 

In an established animal model of retinal oxidative stress, 1 μl of 0.5 mM paraquat was injected into the vitreous cavity of rats. The rats were anesthetized with intraperitoneal 2% pentobarbital (40 mg/kg) and topical 1% proparacaine eye drops. Pupil dilation was achieved with 1% tropicamide. Sclerotomy was performed 1 mm behind the limbus with the tip of a 27 gauge needle. A 33-gauge blunt tip needle (Hamilton, Reno, NV, USA) was inserted into the vitreous cavity and 1 μl of paraquat was injected. The needle was left in the vitreous cavity for 1 min after injection to reduce the degree of reflux. The contralateral eye of each rat was injected with phosphate-buffered saline (PBS) to serve as the control.

### Animal grouping and treatment

SD rats were randomly divided into six groups:

Intravitreous injection of PBS, served as normal control (normal).
Daily intragastrically administered with COS for 14 days.
Intravitreous injection of 0.5 mM paraquat
Intravitreous injection of 0.5 mM paraquat and subsequently daily intragastrically administered with PBS for 14 days.
Intravitreous injection of 0.5 mM paraquat and subsequently daily intragastrically administered with COS at a dose of 5 mg/kg body weight for 14 days.
Intravitreous injection of 0.5 mM paraquat and subsequently daily intragastrically administered with COS at a dose of 10 mg/kg body weight for 14 days.

### Electroretinogram (ERG) recordings

Rats were dark-adapted for 1 hour before performing the ERG. All manipulations were done with dim red light illumination. After being anesthetized, the rats were place on a heating pad. A recording electrode was placed on the cornea after application of 0.5% methyl cellulose. A reference electrode was attached to the shaven skin of the head and a ground electrode clipped the animal’s tail. A single flash light (duration, 100 ms) 30 cm form the eye was used as the light stimulus. Responses were amplified with a gain setting ± 500 μV and filtered with low 0.3 Hz and high 500 Hz from an amplifier. The pattern of the a- and b-wave was recorded. The b-wave ratio was defined as b-wave amplitude of right eye/b-wave amplitude of left eye. The fold of b-wave ratio represented the b-wave ratio at day 3, 7, or 14 /b-wave ratio at day 0.

### Histological study and retinal cell count

For every control and experimental animals, both eyes were collected 14 days after treatment. The specimens were fixed with 4% paraformaldehyde in phosphate buffer saline (PBS, pH 7.4) for 30 min at 4°C, and then washed with PBS. Retinal sections of 5 μm were obtained by cutting along the vertical meridian of the eye and passing through the optic nerve head. The sections were stained with hematoxylin and eosin (H&E) and examined by light microscope (Leica DM 2500; Leica Microsystems, Wetzlar, Germany) with a digital image camera (Leica DFC4200; Leica Microsystem) for morphological observation and retinal cell counts. 

The estimation of retinal rescue was made by comparing the mean number of cells in the inner nuclear layer (INL) and outer nuclear layer (ONL) of the retinas in each treatment group. Retinas approximately 100μm temporal to the midline sagittal cut were sectioned and sampled. Cells numbers in ONL and INL were counted at intervals of 250 μm, starting at approximately 1 mm away from the optic nerve on superior and inferior sides. Six areas of cells were counted and the superior sides were sequentially labeled as area 1 to 3 and inferior sides as area 4 to 6. The result is expressed as the cell number per 250 μm of the retina. Five eyes in each group were used for the experiment.

### Retinal flat-mount and counting of NeuN positive ganglion cell layer neurons

NeuN-positive neurons in the ganglion cell layer, including retinal ganglion cells and displaced amacrine cells, were imaged by microscopy in flat-mounted retinas. Individual retinas were sampled randomly to collect a total of 20 images located at the same eccentricity in the four retinal quadrants using a 20x objective lens. NeuN-positive neurons with a size range of 6 to 30 μm were counted using Image-Pro plus (Media Cybernetics, Bethesda, MD, USA) software. Cell losses in the ischemic retinas were calculated as percentiles of the mean cell density in normal fellow control eyes.

### Terminal deoxynucleotidyl transferase-mediated dUTP-biotinide end labeling (TUNEL)

Rats were euthanized and frozen sections were prepared as described above. TUNEL assays were performed according to the manufacturer’s instructions. Sections were visualized on a fluorescent microscope (Nikon, Melville, NY, USA).

### Measurement of Bax/ Bcl-2 ratio in retinas

Rats were sacrificed at day 3, 7, and 14 after treatment and retinas were isolated and processed for enzyme-linked immunosorbent assay (ELISA). Four isolated retinas of each sample in 500 μl of 0.16 mg/ml heparin saline were centrifuged at 3500 g for 10 min. Sediments were homogenized in 300 μl 20 mM Tris–HCl. The homogenate was first centrifuged twice at 10,000 g for 5 sec. The supernatant was centrifuged again at 3500 g for 15 min. The last supernatant was harvested and frozen at -20°C for subsequent analysis. These samples were analyzed using the Bax ELISA kit (USCNK Life Science Inc., Houston, TX, USA) and Bcl-2 ELISA kit (Cusabio Biotech, Wuhan, China) and according to the manufacturer’s instructions.

### Measurement of reactive oxygen species in retina

Luminol and lucigenin chemiluminenscences (CL) were measured as indicators of free radical formation. Rats were sacrificed at day 3, 7, and 14 and retinas were isolated and then homogenized with PBS. Lucigenin (bis-N-methylacridiniumnitrate) and luminol (5-amino-2,3-dihydro-1,4-phthalazinedione) were obtained from Sigma (St. Louis, MO, USA). Measurements were made at room temperature using a luminescence reader (Aloka Co, Tokyo, Japan). Specimens were put into vials containing PBS-HEPES buffer (0.5 M PBS containing 20 mM HEPES, pH 7.2). ROS were quantified after the addition of enhancers such as lucigenin or luminol (final concentration of 0.2 mM). Counts were obtained at 1 min intervals and the results were given as the area under curve (AUC) for a counting period of 5 min. Counts was corrected for wet tissue weight (rlu/mg tissue).

### Immunohistochemistry

Immunohistochemistry was carried out by simultaneously blocking and permeabilizing sections with 0.2% Triton in PBS containing 5% goat serum for 1 h at room temperature, incubating with primary antibodies diluted in blocking solution overnight at 4°C, and incubating with the appropriate fluorescent secondary antibodies (all diluted 1:1000) in blocking solution for 3 hours at room temperature. Nuclei were counterstained with DAPI. Primary antibodies included GFP (1:5000), cleaved caspase-3, acrolein, nitrotyrosine, 8-Hydroxy-2'-deoxyguanosine (8-OHdG) and NF-κB p65. 

The following formula was used for the densitometric quantitation of acrolein, nitrotyrosine, 8-OHdG and NF-κB p65 immunohistochemistry, as previously described [17] with modification. 

Immunostaining index = Σ [(X-threshold) × area (pixels)] / total cell number

Where X is the staining density indicated by a number between 0 and 256 in grayscale, and X is more than the threshold. Briefly, digitized color images were obtained as PICT files. PICT files were opened in grayscale mode using NIH image, ver. 1.61. Cell numbers were determined using the Analyze Particle command after setting a proper threshold. 

The relative density of immunostaining was defined as immunostaining index of PQ only, COS only, PBS-treated, low-dose or high-dose COS treated groups / immunostaining index of normal group. 

### Measurements of the activities of antioxidant enzymes in retinas

The levels of superoxide dismutase (SOD) and catalase activities in the retina were determined by colorimetric assays at days 3, 7, and 14. Both SOD and catalase assay kits were purchased from Cayman Chemical (Ann Arbor, MI, USA). Assay procedures and tissue homogenate preparations were performed following the manufacturer’s instructions.

### Measurements of glutathione levels in retinas

Glutathione (GSH) ELISA kits were obtained from Cayman (Ann Arbor, MI, USA). The concentration of GSH was determined using the dithionitrobenzoic acid (DTNB) method at 412 nm at days 3, 7, and 14. GSH concentrations were expressed as μmol/mg protein.

### RGC-5 cell culture and oxidative insult in vitro

RGC-5 cells were obtained from Bioresource Collection and Research (Hsinchu, Taiwan) and used for in vitro studies. RGC-5 cells were incubated in Dulbecco's Modified Eagle Media (DMEM) supplemented with 10% (v/v) fetal bovine serum, 500 U/ml penicillin and 500 μg/ml streptomycin. All cells were maintained at 37°C in a humidified 5% CO_2_, 95% air incubator. For the oxidative insult, paraquat was diluted with culture medium to the final concentration of 0.5 mM immediately before use. Cells were seeded on coverslips for 24 hours and treated with 0.5 mM PQ and 50 or 100 μg/ml of COS for another 6 hours. The protocol utilized for TUNEL staining was as described previously. Cells were counterstained with DAPI. The TUNEL assay results were examined by immunofluorescence microscopy.

### Measurement of cell viability

Cell viability was determined by MTT assay after a 12-hour exposure to 0.5 mM PQ and 50 or 100 μg/ml of COS. Briefly, 5 mg/ml of 3-(4,5-Dimethylthiazol-2-yl)-2,5-diphenyl tetrazolium bromide (Chemicon, Temecula, CA, USA) was added to 0.1 ml of cell suspension for 12 hours, and the resulting formazan was then dissolved in isopropanol. Optical density was measured with a plate reader at 570 nm. Cell viability was determined by the MTT reduction assay. 

### Western blot analysis

The rats in each group were sacrificed and retinas were isolated at day 14. In vitro study, RGC-5 cells were stimulated with 0.5 M PQ and then treated with 100 μg/ml COS for 3, 6, 12, and 24 hours. Total protein was extracted from the retinas and the cells by lysing the sample in radioimmunoprecipitation assay (RIPA) buffer (0.5 M Tris-HCl [pH 7.4], 1.5 M NaCl, 2.5% deoxycholic acid, 10% NP-40, 10 mM EDTA and protease inhibitors [Complete Mini; Roche Diagnostics Corp., Indianapolis, IN, USA]). The extract and Laemmli buffer were mixed at a 1:1 ratio, and the mixture was boiled for 5 minutes. Samples (100 μg) were separated on 10% SDS-polyacrylamide gels and then transferred to polyvinylidene difluoride membranes (Immobilon-P; Millipore Corp., Billerica, MA, USA). The membranes were incubated with anti-cleaved caspase-3, anti-IκB, anti-NF-κB p65, and anti-β-actin antibodies. The membranes were subsequently incubated with horseradish peroxidase-conjugated secondary antibody and visualized by chemiluminescence (GE Healthcare, Buckinghamshire, UK). The density of the blots was quantified using image-analysis software after scanning the image (Photoshop, version 7.0; Adobe Systems). The optical densities of each band were calculated and standardized based on the density of the β-actin band. 

### Nuclear Protein Extract and Electrophoretic Mobility Shift Assay of NF-κB (EMSA)

RGC-5 cells were stimulated with 0.5mM PQ and then treated with 50 or 100 μg/ml COS for 3, 6, and 12 hours. The cells were harvested and minced in 0.5 mL of ice-cold buffer A containing 10 mM HEPES (pH 7.9), 1.5 mM KCl, 10 mM MgCl_2_, 1.0 mM dithiothreitol (DTT), and 1.0 mM phenylmethylsulfonyl fluoride (PMSF). The cell suspension was centrifuged at 5000 *g* at 4°C for 10 min. The sediment was suspended in 200 μl of buffer B containing 20 mM HEPES (pH 7.9), 25% glycerol, 1.5 mM MgCl_2_, 420 mM NaCl, 0.5 mM DTT, 0.2 mM EDTA, 0.5 mM PMSF, and 4 μM leupeptin. The suspension was incubated on ice for 30 minutes. The sample was centrifuged at 12,000 *g* at 4°C for 30 minutes. The supernatant containing the nuclear proteins was collected and stored at −70°C until use. The protein concentration was determined with a bicinchoninic acid assay kit with bovine serum albumin (BSA) as the standard (Pierce Biotechnology, Rockford, IL, USA). The EMSA was performed with an NF-κB DNA-binding protein-detection system (Pierce Biotechnology, Rockford, IL, USA) according to the manufacturer's instructions. A 10 μg nuclear protein aliquot was incubated in binding buffer with a biotin-labeled NF-κB consensus oligonucleotide probe (5′-AGTTGAGGGGACTTTCCCAGGC-3′) for 30 minutes. The specificity of the DNA/protein binding was determined by adding a 100-fold molar excess of unlabeled NF-κB oligonucleotide for competitive binding 10 min before adding the biotin-labeled probe. 

### Semi-quantitative polymerase Chain Reaction (PCR)

RGC-5 cells were stimulated with 0.5mM PQ and then treated with 50 μg/ml (low-dose) or 100 μg/ml (high-dose) COS for 12 hours. Total RNA was extracted from RGC-5 cells with Trizol reagent (Invitrogen-Gibco, Grand Island, NY, USA). One microgram of total RNA was annealed for 5 min at 65°C with 300-ng oligo(dT)(Promega, Madison, WI, USA) and reverse transcribed to cDNA using 80 U Moloney murine leukemia virus reverse transcriptase (MMLV-RT) (Invitrogen-Gibco, Grand Island, NY, USA) for 1 h at 37°C. The reaction was stopped by heating at 90°C for 5 min. The cDNA obtained from total RNA was used as a template for PCR amplification. The following primers were used for amplification reaction: for MCP-1, forward primer 5′-GCTCATAGCAGCCACCTT CATTC-3′; reverse primer 5′-GTCTTCGGAGTTTGGGTT TGC-3′; for iNOS, forward primer 5′-TATCTGCAGACACATACTTTACGC-3′; reverse primer 5′-TCCTGGAACCACTCGTACTTG-3′ and for β-actin, forward primer 5′-GAAC CCTAAGGCCAACCGTG-3′; reverse primer 5′-TGGCATAGAGGTCTTTA CGG-3′. The amplification was performed in 30 cycles at 55 °C, 30 s; 72 °C, 1 min and 94 °C, 30 s. PCR products were separated by performing gel electrophoresis on 2% agarose containing ethidium bromide. The intensity of the products was analyzed using an image analyzer (Digital 1D Science; Rochester, NY, USA), and each reverse-transcribed sample was standardized against the amount of β-actin. 

### Statistical analysis

Results are expressed as means ± SD. Statistical analysis for multiple comparisons in each study was determined by the analysis of variance (one- or two-way ANOVA) followed by the Bonferroni analysis. The Student’s t-test was used for pairwise comparisons of the means of two independent groups. A *p* value of 0.05 or less was considered significant. All data were analyzed with the aid of the SPSS 10.0 for Windows statistical package.

## Conclusions

Our studies in a rat model demonstrated that COS attenuates oxidative-stress related retinal degeneration in a dose-dependent manner. The therapeutic effects of COS are probably related to a counteraction of free radicals, recovery of the activities of anti-oxidative enzymes, and inhibition of the activation of NF-κB. COS could be a promising agent for treating oxidative stress-induced retinal diseases. 
